# Diverse Roles of PUF Proteins in Germline Stem and Progenitor Cell Development in *C. elegans*

**DOI:** 10.3389/fcell.2020.00029

**Published:** 2020-02-06

**Authors:** Xiaobo Wang, Ekaterina Voronina

**Affiliations:** Division of Biological Sciences, University of Montana, Missoula, MT, United States

**Keywords:** germline, *C. elegans*, pumilio and *fem-3*-binding factor, RNA regulation, stem cells

## Abstract

Stem cell development depends on post-transcriptional regulation mediated by RNA-binding proteins (RBPs) (Zhang et al., 1997; Forbes and Lehmann, 1998; Okano et al., 2005; Ratti et al., 2006; Kwon et al., 2013). Pumilio and FBF (PUF) family RBPs are highly conserved post-transcriptional regulators that are critical for stem cell maintenance (Wickens et al., 2002; Quenault et al., 2011). The RNA-binding domains of PUF proteins recognize a family of related sequence motifs in the target mRNAs, yet individual PUF proteins have clearly distinct biological functions (Lu et al., 2009; Wang et al., 2018). The *C. elegans* germline is a simple and powerful model system for analyzing regulation of stem cell development. Studies in *C. elegans* uncovered specific physiological roles for PUFs expressed in the germline stem cells ranging from control of proliferation and differentiation to regulation of the sperm/oocyte decision. Importantly, recent studies started to illuminate the mechanisms behind PUF functional divergence. This review summarizes the many roles of PUF-8, FBF-1, and FBF-2 in germline stem and progenitor cells (SPCs) and discusses the factors accounting for their distinct biological functions. PUF proteins are conserved in evolution, and insights into PUF-mediated regulation provided by the *C. elegans* model system are likely relevant for other organisms.

## Introduction

Post-transcriptional regulation of gene expression governs the rate of protein production through the control of key steps in mRNA life cycle. In eukaryotes, RNA-binding proteins (RBPs) play critical roles in mRNA biogenesis, stability, function, transport, and cellular localization essential for post-transcriptional regulation (Glisovic et al., 2008). RBPs expressed in stem cells contribute to the regulation of stem cell self-renewal and differentiation (Zhang et al., 1997; Forbes and Lehmann, 1998; Okano et al., 2005; Ratti et al., 2006; Kwon et al., 2013), while misregulation of RBP activity can lead to tumors (Rezza et al., 2010; Degrauwe et al., 2016). Post-transcriptional regulation in stem cells relies on the combined activities of many RBPs (Eckmann et al., 2004; Arvola et al., 2017). Investigating the basic mechanisms of RBP function in stem cells will advance our understanding of abnormal post-transcriptional regulation relevant to human diseases, such as cancer.

Pumilio and FBF family RBPs are highly conserved eukaryotic posttranscriptional regulators (Wickens et al., 2002; Quenault et al., 2011). The name of this family comes from the first identified PUF proteins, Pumilio in *D. melanogaster* and *fem-3*-binding factor (FBF) in *C. elegans*. PUF proteins control diverse biological processes including oogenesis (Parisi and Lin, 1999), organelle biogenesis (García-Rodríguez et al., 2007), neuronal function (Mee et al., 2004), and memory formation (Dubnau et al., 2003; Zhang et al., 2017). In addition to these roles, PUF proteins share an evolutionarily conserved role in stem cell maintenance. Mutation of Pumilio induces loss of female germline stem cells in *Drosophila* due to differentiation to cystoblasts and then egg chambers (Lin and Spradling, 1997; Forbes and Lehmann, 1998). Similarly, loss of PUF proteins in *C. elegans* results in germline stem cells entering meiosis and undergoing spermatogenesis (Zhang et al., 1997; Crittenden et al., 2002; Haupt et al., 2019b) and knockdown of planarian homolog *DjPum* by RNA interference induces loss of totipotent stem cells called neoblasts (Salvetti et al., 2005). In mammals, PUM proteins contribute to stem cell maintenance across multiple tissues (Shigunov et al., 2012; Naudin et al., 2017; Zhang et al., 2017).

Canonical PUF proteins are characterized by a conserved RNA-binding domain (Pumilio homology domain, PUM-HD) with eight consecutive α-helical PUM repeats (Zamore et al., 1997; Zhang et al., 1997; Wang et al., 2001; Hall, 2016). Crystal structures of the classical PUM-HD uncover a crescent arrangement of PUM repeats. Single-stranded RNA binds to the inner concave surface of PUM-HD. Typically, one PUM repeat contacts one RNA base. A five-amino-acid motif in the second alpha helix of a PUM repeat determines the sequence specificity of RNA base recognition (Wang et al., 2002; Cheong and Hall, 2006; Campbell et al., 2014). Three key residues in the motif directly interact with RNA, thus comprising the tripartite recognition motifs (TRMs) (Wang et al., 2002; Campbell et al., 2014; Hall, 2016). Although individual PUF proteins preferentially associate with RNA motifs of distinct lengths and sequences, the canonical target motifs share the core UGU triplet (Lu et al., 2009; Wang et al., 2018).

Pumilio and FBF proteins control stability and translation of their target mRNAs by binding to their 3’UTRs (Zamore et al., 1997; Zhang et al., 1997). The best-documented mechanism of PUF-mediated regulation is through deadenylation of the target mRNAs that results in translational repression or mRNA decay (Wreden et al., 1997; Goldstrohm et al., 2006; Kadyrova et al., 2007; Van Etten et al., 2012; Weidmann et al., 2014). Alternatively, PUFs can interfere with recognition of cap structure by translation initiation factors through directly binding to the cap (Cao et al., 2010) or through recruiting cap-binding cofactors (Cho et al., 2005, 2006). Additionally, PUFs might attenuate translational elongation through an interaction with Argonaute family proteins (Friend et al., 2012). For all PUFs investigated to date, high-throughput approaches have suggested a large number of putative regulatory targets. Putative PUF-regulated transcripts have been identified in yeast, *Drosophila, C. elegans*, and humans by using RIP (RNA Immunoprecipitation)-Chip, RIP-seq, and CLIP (Cross-linking immunoprecipitation)-seq (Gerber et al., 2004, 2006; Morris et al., 2008; Hafner et al., 2010; Prasad et al., 2016; Porter et al., 2019). The conservation of a number of PUF targets between nematodes and other species including humans was first reported in a microarray study (Kershner and Kimble, 2010) and then confirmed and expanded by CLIP-seq analysis (Prasad et al., 2016; Porter et al., 2019). The shared PUF target mRNAs are enriched for biological process GO terms such as cell cycle, cell division, and nuclear division. Cell cycle regulation is central to stem cell maintenance (Boward et al., 2016), and mRNA target conservation reflects PUF proteins’ ancient role in stem cell maintenance.

The *C. elegans* germline is a powerful model that revealed many aspects of PUF protein function in germline stem cells. Ten PUF proteins identified in *C. elegans* are clustered into 4 subfamilies: PUF-8/9, FBF-1/2, PUF-3/11/4, and PUF-5/6/7 (Wickens et al., 2002; Stumpf et al., 2008; Hubstenberger et al., 2012; Liu et al., 2012). Five of these PUF proteins, FBF-1 and FBF-2, as well as PUF-8, PUF-3, and PUF-11 are enriched in germline stem cells and support stem cell maintenance (Crittenden et al., 2002; Lamont et al., 2004; Ariz et al., 2009; Racher and Hansen, 2012; Voronina et al., 2012; Haupt et al., 2019b), yet each is functionally distinct. In-depth studies of *C. elegans* germline PUF proteins provided novel insights into the mechanisms mediating this functional specialization. This review provides an overview of *C. elegans* germline stem cells and focuses on the contribution of PUF-8, FBF-1, and FBF-2 to germline stem and progenitor cell function, since PUF-3 and PUF-11 are less well-studied. We then discuss recent advances in uncovering the determinants that mediate the divergence of PUF biological functions.

## *C. elegans* Germline, a Powerful Model for Stem Cell Studies

### Overall Structure of *C. elegans* Germline

**FIGURE 1:**
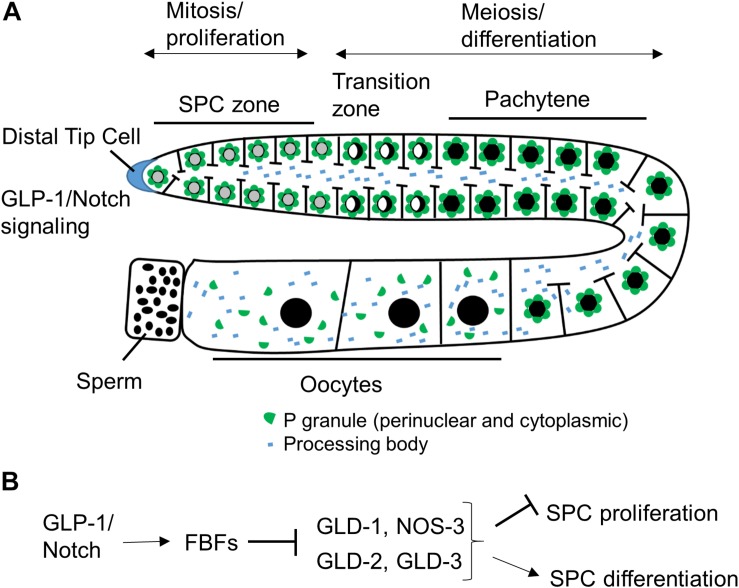
Schematic of *C. elegans* hermaphrodite germline and RNA binding protein network downstream of GLP-1/Notch. **(A)** *C. elegans* germline development is supported by continuous SPC proliferation promoted by GLP-1/Notch signaling from the DTC (Pazdernik and Schedl, 2013). Progenitors enter meiosis when they reach the transition zone, and later differentiate into sperm and oocytes. Several types of RNA granules reside in germ cells and facilitate germ cell development and embryogenesis. **(B)** Downstream of GLP-1/Notch, FBFs maintain SPC proliferation by repressing the expression of GLD-1, GLD-2, and GLD-3 that inhibit SPC proliferation and promote differentiation (Kimble and Crittenden, 2007 and references in sections “RNA-Binding Protein Network Downstream of GLP-1/Notch” and “PUF Function in Maintaining Germline SPCs”).

The *C. elegans* germline is a simple but very powerful model system for studying stem cell biology (Figure 1A). *C. elegans* can exist as hermaphrodites or males, and in this review, we are focusing on hermaphrodites, although mechanisms regulating germline stem cells are similar in the two sexes. A *C. elegans* adult contains two symmetric U-shaped germlines. Most of the *C. elegans* germline, except for late oocytes, is a syncytium, where individual germ cells have an opening to a central shared cytoplasmic core (Hirsh et al., 1976). Although germ cells have access to continuous cytoplasm, the communication between cells is limited and neighboring germ cells can be seen at distinct stages of cell cycle or differentiation. Similar to the germlines of other organisms, the *C. elegans* germline is maintained by a population of proliferative stem cells in the stem cell niche at its distal end (Figure 1A; Pazdernik and Schedl, 2013). When progenitor cells leave the niche, they enter meiosis followed by differentiation into sperm during larval development and into oocytes in adulthood. Maintenance of stem and progenitor cells (SPCs) in the mitotic zone is critical for *C. elegans* germline development and worm fertility.

### Germline Stem and Progenitor Cells

The proliferative zone of the *C. elegans* germline extends about 20 cell diameters from the distal tip, and contains cells in a mitotic cell cycle and cells that have entered meiotic S-phase (Crittenden et al., 2006; Jaramillo-Lambert et al., 2007; Fox et al., 2011). Unlike other stem cell systems with distinct stem cells and transit amplifying cells, the proliferative zone contains developmentally equivalent cells (Fox and Schedl, 2015). In this review, we collectively refer to the cells that have not entered meiosis as SPCs. The *C. elegans* germline SPC zone is maintained within a niche formed by a single mesenchymal cell, called the distal tip cell (DTC), which caps the distal end of the germline and extends its cytoplasmic processes proximally (Kimble and White, 1981; Crittenden et al., 2006; Byrd et al., 2014). The DTC preserves the mitotic identity and promotes mitotic division of SPCs through the canonical GLP-1/Notch signaling that is highly conserved in most multi-cellular organisms (Austin and Kimble, 1987). Loss-of-function mutations of GLP-1/Notch signaling components such as the receptor *glp-1*, ligands *lag-2* and *apx-1*, and downstream transcriptional targets *lst-1* and *sygl-1* cause germline stem cells to enter meiosis prematurely, which is similar to the DTC removal (Kimble and White, 1981; Austin and Kimble, 1987; Henderson et al., 1997; Nadarajan et al., 2009; Kershner et al., 2014). By contrast, germ cells of the *glp-1(oz112gf)* gain-of-function mutant with constitutive GLP-1 signaling fail to exit from the mitotic cell cycle leading to tumorous germlines (Berry et al., 1997).

### RNA-Binding Protein Network Downstream of GLP-1/Notch

Post-transcriptional control is a widespread mechanism for regulating gene expression in the *C. elegans* oogenic germline (Merritt et al., 2008). Downstream of GLP-1/Notch, germline stem cell development is regulated by a network of posttranscriptional regulators that includes a large number of RBPs, a subset of which is shown in Figure 1B. FBF-1 and FBF-2, PUF family RBPs expressed in the distal germline, control stem cell maintenance and sex fate (Zhang et al., 1997; Crittenden et al., 2002). Additionally, four RNA regulators, including three GLD proteins and NOS-3, act in two parallel pathways that inhibit mitosis and promote meiosis (Kimble and Crittenden, 2007). GLD-1 (a KH-motif RBP) and NOS-3 (Nanos protein family member) form a translational repression pathway (Hansen et al., 2004), while the cytoplasmic poly(A) polymerase formed by the complex of GLD-2 [the poly(A) polymerase enzyme] and GLD-3 (a homolog of Bicaudal-C RBP) constitutes a translational activation pathway (Eckmann et al., 2004).

### Cytoplasmic Organization of RNA Regulation

Many RBPs that mediate post-transcriptional regulation of germ cell development are found enriched at cytoplasmic foci called RNA granules. Germ cells have a number of RNA granule subtypes (Figure 1A), including germ granules or P granules in *C. elegans*, processing bodies, and stress granules (Voronina et al., 2011). The processing bodies and stress granules are distributed throughout the cytoplasm in somatic cells as well as in germ cells (Boag et al., 2005; Noble et al., 2008; Hubstenberger et al., 2017; Lechler et al., 2017). By contrast, P granules are perinuclear cytoplasmic RNA granules specific to germ cells and present throughout germline development, excluding mature sperm (Strome and Wood, 1982). All PUF proteins expressed in the *C. elegans* germline are found in RNA granules (Noble et al., 2008; Ariz et al., 2009; Voronina et al., 2012; Haupt et al., 2019b). PUF-5 colocalizes with processing body components (Noble et al., 2008), PUF-8 and FBF-2 localize to P granules (Ariz et al., 2009; Voronina et al., 2012; Wang et al., 2016), and the identities of RNA granules containing FBF-1 or PUF-3 and PUF-11 are currently unknown.

## Regulatory Roles of Puf Proteins in *C. elegans* Germline Stem and Progenitor Cells

### PUF Function in Maintaining Germline SPCs

**FIGURE 2:**
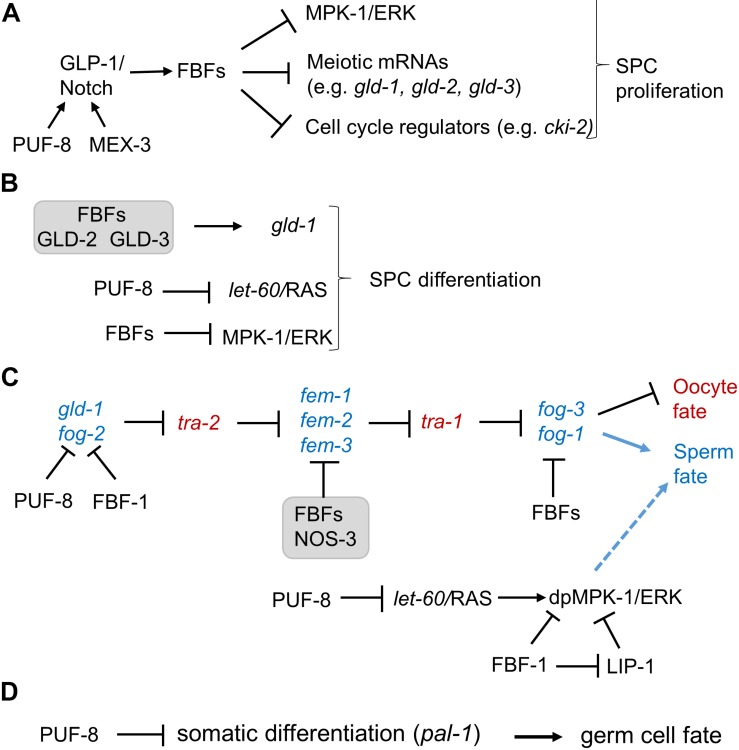
The multiple functions of FBFs and PUF-8 in *C. elegans* germline SPCs. **(A)** PUF-8 acts redundantly with MEX-3 to facilitate GLP-1 signaling (Ariz et al., 2009). Downstream of GLP-1/Notch, FBFs promote germline SPC proliferation by repressing cell cycle regulators, meiotic mRNAs, and *mpk-1* MAP kinase (Crittenden et al., 2002; Lee et al., 2007a; Kalchhauser et al., 2011). **(B)** FBFs act with GLD-2, GLD-3 complex to promote SPC meiosis by activating GLD-1 expression (Suh et al., 2009). PUF-8 facilitates meiosis by repressing LET-60/RAS (Vaid et al., 2013), while FBFs repress *mpk-1*. The contribution of *mpk-1* repression by FBFs to regulation of SPC proliferation and differentiation is discussed in section “PUF Function in Inhibiting Mitotic Cell Fate of SPCs and Promoting Differentiation.” **(C)** PUF-8 controls the sperm/oocyte switch by acting redundantly with FBF-1 to repress *fog-2* (Bachorik and Kimble, 2005). FBF proteins control the sperm/oocyte switch by acting with NOS-3 to repress *fem-3* (Kraemer et al., 1999; Arur et al., 2011) as well as by repressing *fog-1* and possibly *fog-3* (Thompson et al., 2005). Both PUF-8 and FBF-1 cooperate with LIP-1 to repress MPK-1 activity in SPCs, dpMPK-1 refers to a diphosphorylated active form of MPK-1 (discussion and references in section “PUF Function in Controlling the Sperm/Oocyte Decision in Germline Mitotic Zone”). dpMPK-1 promotes spermatogenesis, although specific relevant substrates are yet unknown. **(D)** PUF-8 maintains germ cell fate by repressing somatic transcription factor PAL-1 (Mainpal et al., 2011).

Germline stem cells are maintained by promoting proliferation and/or inhibiting cell death and differentiation. FBF-1 and FBF-2 are redundantly required for maintaining germline SPCs in adult hermaphrodites since a *C. elegans* double mutant for both FBFs lose their germline stem cells by 24 h after the last larval stage (Crittenden et al., 2002). Several FBF targets have been proposed to contribute to FBFs’ role in SPC maintenance (Figure 2A). First, FBFs are suggested to repress expression of MPK-1, a homolog of Mitogen-activated protein kinase (MAPK)/ERK, and *mpk-1* mRNA contains two FBF binding elements in its 3’UTR (Lee et al., 2007a). This repression was hypothesized to be important for stem cell maintenance since RNAi-mediated knockdown of *mpk-1* increased the number of mitotic germ cells, while promoting MPK-1 activity by a Ras gain-of-function mutation *let-60(n1046)* decreased the number of mitotic germ cells (Lee et al., 2006). Similarly, MAPK repression is observed to promote self-renewal of embryonic stem cells and skeletal muscle stem cells (Burdon et al., 1999; Bernet et al., 2014). However, in addition to repressing MPK-1, FBFs repress the expression of its negative regulator, MAPK inactivating phosphatase LIP-1 (Lee et al., 2006). Therefore, an *fbf* mutation would derepress both MPK-1 and LIP-1 that inhibits MAPK activity and thus might not result in abnormal activation of MPK-1 in SPCs. Instead, such mutation would result in a sensitized background that might deregulate MPK-1 following additional genetic lesions. Regulation of MAPK by PUF homologs appears conserved in evolution, and was also documented in human embryonic stem cells as well as in mouse spermatocytes (Lee et al., 2007a; Chen et al., 2012). Second, FBFs promote self-renewal of germline stem cells by repressing expression of CKI-2 (Kalchhauser et al., 2011), a Cyclin-dependent kinase inhibitor that regulates cell cycle entry/exit decisions (Buck et al., 2009). Removing *cki-2* partially rescues the germline stem cell depletion phenotype in *fbf-1 fbf-2* double mutant adult hermaphrodites (Kalchhauser et al., 2011), suggesting that repression of *cki-2* is not the only mechanism by which FBFs promote stem cell proliferation. CIP/KIP family cyclin-dependent kinase inhibitors are conserved targets of PUF proteins as they were found to be regulated by PUFs in mouse embryos and human cells (Kedde et al., 2010; Lin et al., 2019). Interestingly, genes encoding diverse cell cycle regulators, beyond *cki-2* and its homologs, are enriched among target mRNAs pulled down with FBFs as well as PUF proteins from other organisms (Hafner et al., 2010; Kershner and Kimble, 2010; Chen et al., 2012; Prasad et al., 2016; Porter et al., 2019), suggesting a conserved mechanism of PUF-mediated control of cell proliferation. Third, FBFs prevent premature meiotic entry of SPCs by inhibiting expression of target mRNAs that encode differentiation-promoting regulators, such as GLD-1 (Crittenden et al., 2002), GLD-2 (Millonigg et al., 2014), and GLD-3 (Eckmann et al., 2004), as well as structural components of meiotic chromosomes, such as HTP-1,-2 orthologs of human HORMAD1 and 2 (Merritt and Seydoux, 2010).

PUF-8 promotes germline SPC proliferation by acting redundantly with a KH domain-containing RBP MEX-3 (Figure 2A; Ariz et al., 2009). This function might be explained by PUF-8-dependent translational control of cell cycle regulators, but the analysis of GLP-1/Notch receptor in the mutant germlines uncovered mislocalization of GLP-1 protein (Ariz et al., 2009). It appears that PUF-8 facilitates translation of the endoplasmic reticulum protein FARL-11 that is required for GLP-1 membrane localization (Maheshwari et al., 2016), suggesting another potential mechanism of PUF-8 promoting SPC proliferation.

### PUF Function in Inhibiting Mitotic Cell Fate of SPCs and Promoting Differentiation

In addition to facilitating stem cell maintenance, both FBFs and PUF-8 were unexpectedly found to limit stem cell numbers by promoting stem cell exit from mitosis and differentiation (Figure 2B). The GLP-1/Notch signaling within the distal niche maintains the mitotic cell fate of germline SPCs (Hansen and Schedl, 2006; Kimble and Crittenden, 2007). Temperature-sensitive *glp-1(gf)* mutant animals with excessive GLP-1 activity have slightly enhanced proliferation of germline SPCs at the permissive temperature, and produce tumorous germlines at the restrictive temperature (Berry et al., 1997; Pepper et al., 2003; Wang et al., 2012). Interestingly, *puf-8* knockout strongly enhances germ cell over-proliferation of several *glp-1(gf)* mutants at the permissive temperature. This suggests that *puf-8* might inhibit mitotic cell fate of SPCs through negatively regulating the GLP-1/Notch signaling or by functioning parallel to it (Racher and Hansen, 2012). One relevant target mRNA for PUF-8-mediated inhibition of the mitotic cell fate is *C. elegans* RAS homolog LET-60. Loss of *puf-8* promotes accumulation of both endogenous LET-60 and a GFP:H2B reporter under the control of the *let-60* 3’UTR in mitotic germ cells as well as in early meiotic cells, suggesting direct regulation of *let-60* by PUF-8 (Vaid et al., 2013). Increased levels of LET-60 in *puf-8* mutant are not sufficient to ectopically activate MPK-1 in SPCs (Vaid et al., 2013). However, additional loss of LET-60 negative regulator *gap-3* in the *puf-8; gap-3* double mutant leads to activation of MPK-1 throughout the germline, abnormal mitotic proliferation, and tumorous germlines (Vaid et al., 2013). Interestingly, tumor formation in this genetic background was dependent on MAPK signaling and was repressed by RNAi-mediated depletion of MAPK pathway components (Vaid et al., 2013). It appears that MAPK activation doesn’t always cause the proliferative response of SPCs, since the presence of activated MPK-1 in a different double mutant background (*fbf-1; lip-1*) fails to elicit abnormal proliferation (Lee et al., 2007a). This brings up a question whether MAPK signaling promotes SPC proliferation (Lee et al., 2006) or differentiation (Vaid et al., 2013). Analysis of null mutants in *lin-45*/RAF, *mek-2*/MEK, and *mpk-1*/ERK suggested that MAPK components are not essential for SPC maintenance, but each leads to a decrease in SPC numbers especially as animals age (Lee et al., 2007b). Additionally, null mutants in *lin-45*/RAF, *mek-2*/MEK, and *mpk-1*/ERK enhance premature meiotic entry defect of a temperature-sensitive *glp-1* loss-of-function allele at the permissive temperature, suggesting that MAPK signaling promotes SPC proliferation (Lee et al., 2007b). On the other hand, RNAi depletion of *mpk-1* increased SPC numbers (Lee et al., 2006). The null mutations and RNAi treatment might not affect gene function with the same efficiency, and disparate results obtained by the two approaches might point to the critical differences in specific levels and developmental dynamics of MAPK activity underlying each phenotype. Considering this, regulation of multiple genes affecting the levels of MPK-1-mediated signaling by FBFs and PUF-8 (Lee et al., 2006, 2007a; Vaid et al., 2013) might allow SPCs to maintain precise control of MPK-1 activity during development.

Genetic evidence suggests that FBFs act to promote meiotic entry of SPCs through the GLD-2, GLD-3 genetic pathway (Crittenden et al., 2002). GLD-1, NOS-3 and GLD-2, GLD-3 are the two main pathways that redundantly promote SPC meiotic entry (Figure 1B; Kimble and Crittenden, 2007). In the absence of *gld-1*, FBFs are no longer required to sustain germline proliferation and the *gld-1; fbf-1 fbf-2* mutant worms have tumorous germline with all mitotic cells (Crittenden et al., 2002). This tumorous germline phenotype is similar to the tumors observed in *gld-1; gld-2* and *gld-1; gld-3* mutants (Kadyk and Kimble, 1998; Eckmann et al., 2004), suggesting a possibility that FBF proteins function through the GLD-2, GLD-3 genetic pathway to promote meiotic entry. Direct interaction of FBF with GLD-3 that might underlie this function is discussed further in section “Protein Cofactors That Change PUF Regulatory Outcome.”

The fact that PUF proteins appear to regulate both proliferation and differentiation is enigmatic and has promoted several interpretations. For example, PUF-8 represses some mRNAs associated with proliferation while facilitating expression of other targets promoting proliferation in the same cells. As a result, it is possible that the overall effect of PUF-8 on germline proliferation is minor, and it acts to fine-tune SPC proliferation rather than as an all-or-none switch specifying stem cell fate. In a similar vein, functional annotation of mRNAs co-isolated with FBFs suggests that they associate with and repress mRNAs required for both differentiation as well as cell cycle progression of germ cells (Prasad et al., 2016; Porter et al., 2019). One intriguing interpretation is that this allows FBFs to simultaneously control the rate of both SPC proliferation and differentiation, thus maintaining the balance between these two cell fates. In order to maintain stem cell numbers over time, their self-renewal needs to be matched with differentiation (Morrison and Kimble, 2006). In *C. elegans*, SPC homeostasis is controlled at a population level, where some progenitor cells are lost through differentiation, while other cells proliferate, with both outcomes observed at the same frequency (Kimble and Crittenden, 2007). Although *C. elegans* SPCs proliferate continuously, the rate of SPC proliferation changes during development and is responsive to environmental conditions and nutrition (Hubbard et al., 2013). Simultaneous control of SPC proliferation and differentiation by FBFs might work to match the output of the stem cell compartment to the proliferative demands of the germline, while keeping the two fates in a balance.

### PUF Function in Controlling the Sperm/Oocyte Decision in Germline Mitotic Zone

The mechanism underlying sperm/oocyte decision has been a long-standing question in all animals (Casper and Van Doren, 2006; Kimble and Page, 2007). In *C. elegans* hermaphrodites, germlines first produce sperm and then oocytes, but it is still not clear when, where, and how the sperm/oocyte switch is executed. As recently reviewed (Zanetti and Puoti, 2013), the germline sex determination is executed through an elaborate pathway involving more than 30 regulators for sperm or oocyte specification, part of which is shown in Figure 2C. These regulators, including GLD-1, TRA-1 (GLI transcription factor homolog; Hodgkin, 1987), and FOG-1 (feminization of the germline, a member of cytoplasmic polyadenylation element binding protein family; Jin et al., 2001) are expressed in the proximal mitotic region and transition zone, suggesting that the commitment of germ cells to the sperm or oocyte fate might occur in distal germline. Studies of sex determination in a temperature-sensitive *fog-1* mutant suggested that germ cells might become committed to the sperm or oocyte fate when they enter meiosis (Barton and Kimble, 1990). Further analysis of sex determination in *puf-8; lip-1* worms that permit chemical manipulation of the sperm/oocyte decision supported these earlier conclusions by mapping the sex fate determination to the progenitor cells moving proximally to transition zone (Morgan et al., 2013). PUF-8, FBF-1, and FBF-2 contribute to the control of the sperm/oocyte decision by regulating expression of sex-determination regulators (Figure 2C).

FBF-1 and FBF-2 are redundantly required for controlling the sperm/oocyte switch. Nematodes mutant for individual *fbf* genes produce both sperm and oocytes, but the *fbf* double mutants fail to switch from spermatogenesis to oogenesis and only produce sperm (Zhang et al., 1997). The two FBF paralogs promote oogenesis by repressing several target mRNAs including *fem-3, fog-1*, and possibly *fog-3* that are positive regulators for sperm fate decision (Zhang et al., 1997; Thompson et al., 2005). Additionally, Nanos homolog NOS-3 physically interacts with FBF proteins and participates in the FBF-mediated sperm/oocyte switch through forming a regulatory complex that represses the translation of *fem-3* mRNA (Kraemer et al., 1999; Arur et al., 2011). The binding between NOS-3 and FBF-1 is disrupted by MPK-1/ERK-dependent phosphorylation of NOS-3 to limit formation of the functional complex to the distal germline (Arur et al., 2011). Lastly, functional splicing machinery promotes efficient sperm/oocyte switch (Kerins et al., 2010), and a combination of *fbf* single mutants and splicing factor knockdown results in enhanced germline masculinization, suggesting that the splicing machinery facilitates FBF function in sex determination (Novak et al., 2015).

PUF-8 and FBF-1 also redundantly promote the germline sperm/oocyte switch (Bachorik and Kimble, 2005). A mutation in *puf-8* results in a low percentage of germlines that develop excess sperm and fail to switch to oogenesis, whereas most of the *fbf-1 puf-8* double mutants result in germlines with a failed sperm/oocyte switch. GLD-1 and FOG-2 proteins can physically interact (Clifford et al., 2000), and both are required for the sperm fate determination (Jan et al., 1999; Clifford et al., 2000; Hu et al., 2019). The dramatic increase in FOG-2 protein abundance in *fbf-1 puf-8* double mutants and rescue of oogenesis in *fbf-1 puf-8; fog-2* triple mutants suggests that FBF-1 and PUF-8 function upstream of FOG-2 in the sex determination pathway (Bachorik and Kimble, 2005). In addition, PUF-8 acts redundantly with MEX-3 to promote the sperm/oocyte switch (Ariz et al., 2009). Although *puf-8; mex-3* mutant germlines have severe proliferation defects and never produce any gametes, 34% of *puf-8; mex-3*(+/-) mutant worms produce only sperm (Ariz et al., 2009). This suggests that MEX-3 contributes to the sperm/oocyte switch in the absence of PUF-8, although the relevant regulatory targets have not yet been identified (Ariz et al., 2009).

One of the many functions of MAPK/ERK signaling pathway in *C. elegans* is to promote the sperm fate (Lee et al., 2007b). Therefore, regulation of MPK-1 activity by PUF-8 and FBF-1 reviewed above contributes to germline sex determination. Hyperactivation of MPK-1 and excessive spermatogenesis were observed in *puf-8; lip-1* as well as in *fbf-1; lip-1* genetic backgrounds (Morgan et al., 2010; Sorokin et al., 2014). In these genetic backgrounds, spermatogenesis was dependent on MPK-1 activity and repressed by a small molecule MEK inhibitor U0126 (Morgan et al., 2010; Sorokin et al., 2014). Activation of MPK-1 in *fbf-1; lip-1* genetic background likely results from the loss of FBF-mediated repression of *mpk-1* translation and the loss of LIP-1-mediated post-translational inhibition of MPK-1 (Lee et al., 2007a). On the other hand, PUF-8 limits ERK activity by repressing LET-60/RAS (Vaid et al., 2013), and the *puf-8; lip-1* double mutant results in hyperactivation of MPK-1/ERK at meiotic entry in the transition zone (Morgan et al., 2013).

### PUF-8 Function in Protecting Germ Cell Fate

In multicellular animals, diverse factors and mechanisms, including posttranscriptional regulation, contribute to the maintenance of germ cell fate and protect germ cells from reprograming toward somatic cells (Strome and Updike, 2015). To protect germ cell identity, PUF-8 suppresses the expression of *pal-1* in germline stem cells of *C. elegans* (Figure 2D; Mainpal et al., 2011). PAL-1 is a somatic homeodomain transcription factor that activates transcription of its downstream targets such as *hlh-1* (Hunter and Kenyon, 1996; Lei et al., 2009). In turn, *hlh-1* encodes the myogenic regulatory factor HLH-1/MyoD homolog that is normally expressed in the embryonic muscle lineage (Krause et al., 1990). Depletion of *puf-8* results in derepression of PAL-1 in germline SPCs and PAL-1-dependent misexpression of HLH-1 in germ cells (Mainpal et al., 2011). These findings suggest that PUF-8 protects germline SPCs from the impact of somatic differentiation factors such as PAL-1.

## Mechanisms Behind Functional Divergence of Puf Proteins

The highly conserved RNA-binding domain of canonical PUF family proteins recognizes stereotypical consensus binding sites in target mRNAs (Wang et al., 2018). Yet, as reviewed in the previous section, individual PUF proteins have clearly distinct regulatory functions. In *C. elegans* germline stem cells, activities of multiple PUF proteins combine to promote many aspects of healthy stem cell function. This made *C. elegans* germline an excellent model to probe the mechanisms mediating functional specialization of PUFs. Here we will survey the recent insights into the mechanisms specifying unique non-redundant aspects of RNA regulation mediated by FBF-1, FBF-2, and PUF-8.

### Structural Differences in RNA-Binding Domains Determine the Specificity of Binding RNA

All canonical PUF proteins contain a highly conserved RNA-binding domain, PUF domain (also known as PUM-HD), with eight consecutive α-helical repeats. Crystal structures of the PUM-HDs from different organisms bound to short target RNA motifs revealed that the PUM-HD forms a crescent shape molecule with eight α-helical repeats (Edwards et al., 2001; Wang et al., 2001, 2002, 2009; Zhu et al., 2009). Mutational analysis of *Drosophila* Pumilio revealed the amino acids mediating contacts with the mRNA and protein partners (Zamore et al., 1997; Wharton et al., 1998; Sonoda and Wharton, 1999, 2001). Subsequent structural studies of *Drosophila*, *C. elegans*, and mammalian PUFs extended the genetic and biochemical data and have identified the TRMs that contact RNA on the concave surface as well as the sites on the convex surface that interact with protein partners (Edwards et al., 2001; Wang et al., 2002, 2009; Campbell et al., 2014; Bhat et al., 2019; Qiu et al., 2019). Differences in the PUF RNA-binding domains result in distinct RNA motifs bound by PUF homologs.

*In vitro* biochemical studies using isolated PUF domains found that *C. elegans* FBFs bind to the same RNA motif, a 9-nt motif (5′-UGURHHAUA-3′; Bernstein et al., 2005; Opperman et al., 2005; Campbell et al., 2012), while PUF-8 recognizes an 8-nt motif (5′-UGUANAUA-3′; Opperman et al., 2005; Bhat et al., 2019). Crystal structures of FBF and PUF-8 PUM-HDs in complex with their preferred RNA oligonucleotides uncovered RNA-binding modes for each protein. FBF’s PUM repeats R8-R6 bind to the 5′-UGU sequence and PUM repeats R1–R3 bind to the AUA-3′ element. The purine in the fourth position is recognized by PUM repeat R4, while bases in positions five and six turn away from the RNA-binding surface. Interactions between base five and the protein depend on the identities of the fourth and fifth bases, and base six does not interact with PUM-HD at all (Wang et al., 2009). By comparison, PUF-8’s PUM repeats R8-R5 bind to the 5′-UGUA sequence, while PUM repeats R3-R1 bind to the AUA-3′ sequence with central fifth base stacked with the fourth base and not recognized by the protein (Bhat et al., 2019). Distinct binding site preferences between FBFs and PUF-8 are expected to result in specific mRNA populations associated with these proteins. FBF-1 and FBF-2 share most of their target mRNAs, which has been demonstrated by immunoprecipitation followed by CLIP-seq analyses (Prasad et al., 2016; Porter et al., 2019). Initial characterization of PUF-8 target mRNAs was carried out through a pull-down with recombinant protein followed by micro-array analysis (Mainpal et al., 2011). Although PUF-8 target data is less extensive than those available for FBFs, several notable observations emerge. A number of PUF-8 targets are also present in FBF target lists and some, such as *ubc-6*, are regulated by both PUF-8 and FBFs (Mainpal et al., 2011; Prasad et al., 2016). However, overall the mRNAs bound to PUF-8 and FBFs are largely distinct. While further studies will determine the extent of PUF-8’s targets overlapping with those of FBFs, these initial results provide an attractive model of specifying distinct but redundant functions of FBFs and PUF-8 in the germline.

If distinct binding preferences of PUF proteins underlie the differences in their function, one might expect to elicit functional changes in PUFs through changing the RNA-binding interface. Recent structural study revealed that the RNA-binding preference of FBF-2 can be changed to become similar to that of PUF-8 through mutations in TRM of PUM repeat R5 (Bhat et al., 2019). The FBF-2 R5 variant was tested for its ability to mediate a PUF-8-specific function, namely promoting SPC differentiation in a genetic background of a temperature-sensitive *glp-1(gf)* mutation. While 98% of *glp-1(gf)* germlines with the wild-type FBF-2 developed tumors upon *puf-8* knockdown, over-proliferation was only observed in 36% germlines expressing FBF-2 R5 variant (Bhat et al., 2019). This partial rescue supports the importance of PUF domain RNA-binding preference in specifying function, but it still remains to be determined whether FBF-2 R5 variant truly elicits its new effect through associating with and regulating PUF-8 targets *in vivo*.

### Protein Cofactors That Change RNA Target Preference

**FIGURE 3:**
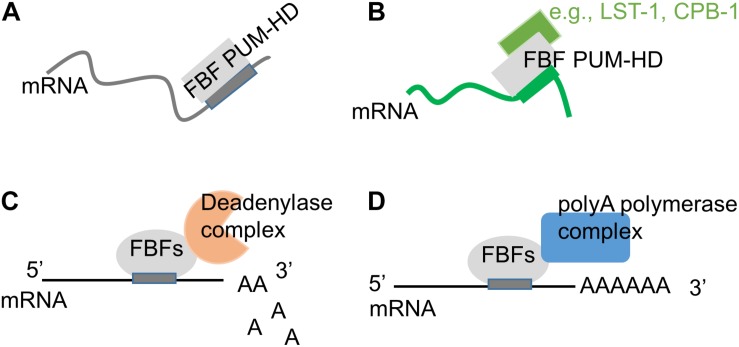
Modification of FBF biological activity though interactions with protein partners. **(A)** On its own, FBF PUF domain binds to target mRNAs containing a canonical 9-nt motif (Wang et al., 2009; Bhat et al., 2019; Qiu et al., 2019). **(B)** FBF PUF domain’s RNA-binding specificity can be influenced by interactions with protein partners such as CPB-1 (Menichelli et al., 2013) and LST-1 (Qiu et al., 2019). **(C)** FBFs can repress target mRNAs by recruiting deadenylase complex (Goldstrohm et al., 2006; Suh et al., 2009). **(D)** FBFs can promote mRNA polyadenylation by interacting with the poly(A) polymerase complex (Eckmann et al., 2002; Suh et al., 2009).

While determination of *in vivo* FBF targets confirmed FBF preferential association with mRNAs containing canonical FBF-binding element identified *in vitro*, many of the identified targets did not contain the canonical motif, suggesting that FBF binding specificity may be altered *in vivo* (Prasad et al., 2016; Porter et al., 2019). Biochemical and structural studies of PUFs in complex with their partner proteins revealed that several PUF interacting partners can affect the RNA-binding affinity and specificity of PUF proteins (Figures 3A,B). Crystal structure of Nanos-Pumilio-RNA complex from *Drosophila* suggested that Nanos embraces Pumilio and RNA, contributes to sequence-specific contacts, and increases Pumilio affinity for *hunchback* mRNA (Weidmann et al., 2016; Malik et al., 2019). By contrast, association of Pumilio with *mothers against dpp (mad)* mRNA requires Bam and Bgcn proteins, but not Nanos (Malik et al., 2019). In *C. elegans*, both FBF proteins physically interact with CPB-1, a cytoplasmic polyadenylation element binding protein (Luitjens et al., 2000; Menichelli et al., 2013). The assay investigating binding of FBF-2 PUF domain to target mRNA in the presence of a 40-amino-acid fragment of CPB-1 outside of the RNA-binding domain demonstrated that association with CPB-1 fragment alters FBF’s preference for specific RNA sequences (Campbell et al., 2012; Menichelli et al., 2013). Additional FBF interaction partners include novel proteins SYGL-1 and LST-1 that are required for FBF-dependent target mRNA repression in germline SPCs (Kershner and Kimble, 2010; Brenner and Schedl, 2016; Shin et al., 2017; Haupt et al., 2019a). Using SEQRS (*in vitro* selection, high-throughput sequencing of RNA, and sequence specificity landscapes), analysis of RNA-binding preference of FBF-2 PUF domain bound to a 150-amino-acid LST-1 fragment containing one of FBF-binding sites revealed a distinct RNA-binding specificity of the FBF-2/LST-1 complex (Qiu et al., 2019). Crystal structure of FBF-2 in complex with a 24-amino-acid fragment of LST-1 and an 8-nucleotide RNA oligo isolated by *in vitro* selection showed that FBF-2 PUF domain changes its RNA-binding mode to 1:1 association of PUM repeats R4-R5 with GA in positions four and five (Qiu et al., 2019). However, the structural basis for the changes in the RNA-binding specificity is not entirely clear since association with LST-1 peptide appeared to weaken FBF-2 affinity for all tested target sequences (Qiu et al., 2019). Further studies are necessary to understand whether association with full-length LST-1 has similar effects on FBF-2 binding to its targets.

### Protein Cofactors That Change PUF Regulatory Outcome

Pumilio and FBF proteins lack enzymatic activity and often mediate their regulatory influence by recruiting specific cofactors to their target mRNAs (Sonoda and Wharton, 1999, 2001; Eckmann et al., 2002; Cho et al., 2005, 2006; Goldstrohm et al., 2006; Kadyrova et al., 2007; Suh et al., 2009; Friend et al., 2012). PUF proteins typically reduce expression of their targets by repressing translation or promoting RNA decay (Wreden et al., 1997; Olivas and Parker, 2000; Crittenden et al., 2002; Goldstrohm et al., 2006; Cao et al., 2010; Weidmann and Goldstrohm, 2012). This repressive function of PUF proteins in *C. elegans* and other species can be mediated by CCR4-NOT deadenylase that promotes RNA deadenylation and decay (Figure 3C), and FBF-1, FBF-2, and PUF-8 all bind deadenylase enzyme CCF-1 (Goldstrohm et al., 2006; Suh et al., 2009). One alternative repressive mechanism suggested for FBFs relies on PUF domain interaction with Argonautes resulting in attenuated translational elongation (Friend et al., 2012).

In several cases, PUF proteins appear to activate translation: FBFs are suggested to promote GLD-1 expression in spermatogenic germline as well as translation of EGL-4 in neurons, while PUF-8 facilitates translation of FARL-11 in germline SPCs (Kaye et al., 2009; Suh et al., 2009; Maheshwari et al., 2016). A search for cofactors of FBFs uncovered an interaction with poly(A) polymerase complex identifying one potential mechanism for translational activation (Figure 3D; Eckmann et al., 2002; Kimble and Crittenden, 2007). FBFs interact with the GLD-3 subunit of GLD-3/GLD-2 cytoplasmic poly(A) polymerase complex (Eckmann et al., 2002). FBFs also interact with the GLD-2 subunit in the RNA-independent manner, and this interaction is facilitated by formation of a larger complex including GLD-3 (Suh et al., 2009). Interaction with GLD-3 does not affect FBFs binding to their target mRNA, and is instead hypothesized to switch the regulatory outcome from repression to activation (Wu et al., 2013).

It is still unknown what cofactors are required for PUF-8-mediated translational activation. Since FBF interacts with GLD-3 via its conserved RNA-binding domain (Wu et al., 2013), it is possible that PUF-8 RNA-binding domain might interact with GLD-3 as well. Additionally, a recent study found that PUF-8 promotes accumulation of several of its target mRNAs through interaction with mRNA processing/export machinery components, such as the nuclear cap-binding protein NCBP-2 (Pushpa et al., 2013).

### Distinct PUF Localization

FBF-1 and FBF-2 are nearly identical in primary sequence, share most of the target mRNAs (Prasad et al., 2016; Porter et al., 2019), and function redundantly in maintaining germline SPCs. Nevertheless, they differentially affect germline SPC zone size as *fbf-2* mutant maintains a larger SPC zone than the *fbf-1* mutant (Lamont et al., 2004). In addition, FBF homologs have different effects on their target mRNAs: FBF-1 promotes the clearance of target mRNAs required for meiosis out of the mitotic region, whereas FBF-2 sequesters target mRNAs while preventing their translation (Voronina et al., 2012). These differences correlate with FBFs’ localization to distinct RNA granules. FBF-2 localizes to P granules and requires P granule integrity for its activity, while FBF-1 localizes to perinuclear RNA granules adjacent to P granules and its activity does not require P granule integrity (Voronina et al., 2012). P granule localization of FBF-2 requires interaction with a small protein DLC-1, dynein light chain 1 (Wang et al., 2016). DLC-1 directly interacts with FBF-2, but not with FBF-1, by binding to several sites outside of FBF-2 RNA-binding domain where FBF-1 and FBF-2 sequences diverge (Wang et al., 2016).

Similar to FBF-2, PUF-8 localizes to P granules as determined by co-immunostaining of PUF-8:GFP and P granule component PGL-1 (Ariz et al., 2009). However, the requirement of P granules for PUF-8 function has not been evaluated so far. Additionally, PUF-8 has been shown to localize to the nuclear cortex, where it has been proposed to interact with the nuclear mRNA export machinery and promote the export of several germline mRNAs (Pushpa et al., 2013).

## Conclusion

Pumilio and FBF family RBPs have evolved as essential post-transcriptional regulators of stem cell development in eukaryotes. PUF-mediated RNA regulation is achieved through recognizing target mRNAs and subsequently changing their rates of degradation or translation. Three PUF proteins, PUF-8, FBF-1 and FBF-2, expressed in *C. elegans* germline mitotic region are required for many aspects of germline SPCs development, and each facilitates specific aspects of SPC function. Studies in *C. elegans* resulted in considerable advances in understanding the mechanisms behind diverse biological activities of PUFs as shown in Figure 3. The next challenge to the field is to uncover the mechanisms directing PUF protein’s choice of specific cofactors and influencing PUFs’ function as negative or positive translational regulators in stem cells.

*fem-3*-binding factors’ association with CPB-1 and LST-1 affects FBF affinity and selectivity toward their target mRNAs. CPB-1 and LST-1 are expressed at different developmental stages, with CPB-1 expressed in differentiating spermatogenic cells (Luitjens et al., 2000) and LST-1 expressed in stem cells (Shin et al., 2017). Their stage-specific association with FBFs might result in a shifting repertoire of FBF regulatory targets across development. Both CPB-1 and LST-1 appear to bind to the same site on FBF RNA-binding domain. Interestingly, this binding site is also shared by GLD-3, a protein that doesn’t affect FBF target selection, but rather might change FBF regulatory outcome from translational repression to translational activation. Since GLD-3 becomes expressed as SPCs transition to meiosis, it is unclear whether GLD-3 competes with LST-1 for binding to FBFs. In the future, it would be important to understand the mechanisms regulating PUF association with their cofactors. In yeast, nutrient-responsive phosphorylation of PUF protein Puf3p at the N-terminal low complexity region can switch the fate of its target mRNAs from degradation to translation (Lee and Tu, 2015), suggesting a possibility that post-translational modifications can provide an additional layer of regulation that affects PUF protein activity.

## Author Contributions

XW and EV contributed equally to writing of the manuscript.

## Conflict of Interest

The authors declare that the research was conducted in the absence of any commercial or financial relationships that could be construed as a potential conflict of interest.

## References

[B1] Ariz M. Mainpal R. Subramaniam K. (2009). *C. elegans* RNA-binding proteins PUF-8 and MEX-3 function redundantly to promote germline stem cell mitosis. *Dev. Biol.* 326 295–304. 10.1016/j.ydbio.2008.11.024 19100255 PMC2680957

[B2] Arur S. Ohmachi M. Berkseth M. Nayak S. Hansen D. Zarkower D. (2011). MPK-1 ERK controls membrane organization in *C. elegans* oogenesis via a sex-determination module. *Dev. Cell* 20 677–688. 10.1016/j.devcel.2011.04.009 21571224 PMC3098718

[B3] Arvola R. M. Weidmann C. A. Tanaka Hall T. M. Goldstrohm A. C. (2017). Combinatorial control of messenger RNAs by Pumilio, Nanos and brain tumor proteins. *RNA Biol.* 14 1445–1456. 10.1080/15476286.2017.1306168 28318367 PMC5785226

[B4] Austin J. Kimble J. (1987). glp-1 is required in the germ line for regulation of the decision between mitosis and meiosis in *C. elegans*. *Cell* 51 589–599. 3677168 10.1016/0092-8674(87)90128-0

[B5] Bachorik J. L. Kimble J. (2005). Redundant control of the *Caenorhabditis elegans* sperm/oocyte switch by PUF-8 and FBF-1, two distinct PUF RNA-binding proteins. *Proc. Natl. Acad. Sci. U.S.A.* 102 10893–10897. 16037210 10.1073/pnas.0504593102 PMC1182444

[B6] Barton M. K. Kimble J. (1990). fog-1, a regulatory gene required for specification of spermatogenesis in the germ line of *Caenorhabditis elegans*. *Genetics* 125 29–39. 2341035 10.1093/genetics/125.1.29 PMC1204007

[B7] Bernet J. D. Doles J. D. Hall J. K. Kelly Tanaka K. Carter T. A. Olwin B. B. (2014). p38 MAPK signaling underlies a cell-autonomous loss of stem cell self-renewal in skeletal muscle of aged mice. *Nat. Med.* 20 265–271. 10.1038/nm.3465 24531379 PMC4070883

[B8] Bernstein D. Hook B. Hajarnavis A. Opperman L. Wickens M. (2005). Binding specificity and mRNA targets of a *C. elegans* PUF protein, FBF-1. *RNA* 11 447–458. 15769874 10.1261/rna.7255805 PMC1370734

[B9] Berry L. W. Westlund B. Schedl T. (1997). Germ-line tumor formation caused by activation of glp-1, a *Caenorhabditis elegans* member of the Notch family of receptors. *Development* 124 925–936. 9043073 10.1242/dev.124.4.925

[B10] Bhat V. D. Mccann K. L. Wang Y. Fonseca D. R. Shukla T. Alexander J. C. (2019). Engineering a conserved RNA regulatory protein repurposes its biological function. *eLife* 8:e43788. 10.7554/eLife.43788 30652968 PMC6351103

[B11] Boag P. R. Nakamura A. Blackwell T. K. (2005). A conserved RNA-protein complex component involved in physiological germline apoptosis regulation in *C. elegans*. *Development* 132 4975–4986. 16221731 10.1242/dev.02060

[B12] Boward B. Wu T. Dalton S. (2016). Concise review: control of cell fate through cell cycle and pluripotency networks. *Stem Cells* 34 1427–1436. 10.1002/stem.2345 26889666 PMC5201256

[B13] Brenner J. L. Schedl T. (2016). Germline stem cell differentiation entails regional control of cell fate regulator GLD-1 in *Caenorhabditis elegans*. *Genetics* 202 1085–1103. 10.1534/genetics.115.185678 26757772 PMC4788111

[B14] Buck S. H. Chiu D. Saito R. M. (2009). The cyclin-dependent kinase inhibitors, cki-1 and cki-2, act in overlapping but distinct pathways to control cell cycle quiescence during *C. elegans* development. *Cell Cycle* 8 2613–2620. 19597327 10.4161/cc.8.16.9354 PMC3141283

[B15] Burdon T. Stracey C. Chambers I. Nichols J. Smith A. (1999). Suppression of SHP-2 and ERK signalling promotes self-renewal of mouse embryonic stem cells. *Dev. Biol.* 210 30–43. 10364425 10.1006/dbio.1999.9265

[B16] Byrd D. T. Knobel K. Affeldt K. Crittenden S. L. Kimble J. (2014). A DTC niche plexus surrounds the germline stem cell pool in *Caenorhabditis elegans*. *PLoS One* 9:e88372. 10.1371/journal.pone.0088372 24586318 PMC3929564

[B17] Campbell Z. T. Bhimsaria D. Valley C. T. Rodriguez-Martinez J. A. Menichelli E. Williamson J. R. (2012). Cooperativity in RNA-protein interactions: global analysis of RNA binding specificity. *Cell Rep.* 1 570–581. 22708079 10.1016/j.celrep.2012.04.003 PMC3375920

[B18] Campbell Z. T. Valley C. T. Wickens M. (2014). A protein-RNA specificity code enables targeted activation of an endogenous human transcript. *Nat. Struct. Mol. Biol.* 21 732–738. 10.1038/nsmb.2847 24997599 PMC4125476

[B19] Cao Q. Padmanabhan K. Richter J. D. (2010). Pumilio 2 controls translation by competing with eIF4E for 7-methyl guanosine cap recognition. *RNA* 16 221–227. 10.1261/rna.1884610 19933321 PMC2802031

[B20] Casper A. Van Doren M. (2006). The control of sexual identity in the *Drosophila* germline. *Development* 133 2783–2791. 16835435 10.1242/dev.02415

[B21] Chen D. Zheng W. Lin A. Uyhazi K. Zhao H. Lin H. (2012). Pumilio 1 suppresses multiple activators of p53 to safeguard spermatogenesis. *Curr. Biol.* 22 420–425. 10.1016/j.cub.2012.01.039 22342750 PMC3449084

[B22] Cheong C. G. Hall T. M. (2006). Engineering RNA sequence specificity of Pumilio repeats. *Proc. Natl. Acad. Sci. U.S.A.* 103 13635–13639. 16954190 10.1073/pnas.0606294103 PMC1564246

[B23] Cho P. F. Gamberi C. Cho-Park Y. A. Cho-Park I. B. Lasko P. Sonenberg N. (2006). Cap-dependent translational inhibition establishes two opposing morphogen gradients in *Drosophila* embryos. *Curr. Biol.* 16 2035–2041. 17055983 10.1016/j.cub.2006.08.093 PMC2238800

[B24] Cho P. F. Poulin F. Cho-Park Y. A. Cho-Park I. B. Chicoine J. D. Lasko P. (2005). A new paradigm for translational control: inhibition via 5′-3′ mRNA tethering by Bicoid and the eIF4E cognate 4EHP. *Cell* 121 411–423. 15882623 10.1016/j.cell.2005.02.024

[B25] Clifford R. Lee M. H. Nayak S. Ohmachi M. Giorgini F. Schedl T. (2000). FOG-2, a novel F-box containing protein, associates with the GLD-1 RNA binding protein and directs male sex determination in the *C. elegans* hermaphrodite germline. *Development* 127 5265–5276. 11076749 10.1242/dev.127.24.5265

[B26] Crittenden S. L. Bernstein D. S. Bachorik J. L. Thompson B. E. Gallegos M. Petcherski A. G. (2002). A conserved RNA-binding protein controls germline stem cells in *Caenorhabditis elegans*. *Nature* 417 660–663. 12050669 10.1038/nature754

[B27] Crittenden S. L. Leonhard K. A. Byrd D. T. Kimble J. (2006). Cellular analyses of the mitotic region in the *Caenorhabditis elegans* adult germ line. *Mol. Biol. Cell* 17 3051–3061. 16672375 10.1091/mbc.E06-03-0170 PMC1552046

[B28] Degrauwe N. Suvà M. L. Janiszewska M. Riggi N. Stamenkovic I. (2016). IMPs: an RNA-binding protein family that provides a link between stem cell maintenance in normal development and cancer. *Genes Dev.* 30 2459–2474. 27940961 10.1101/gad.287540.116 PMC5159662

[B29] Dubnau J. Chiang A. S. Grady L. Barditch J. Gossweiler S. Mcneil J. (2003). The *staufen/pumilio* pathway is involved in *Drosophila* long-term memory. *Curr. Biol.* 13 286–296. 12593794 10.1016/s0960-9822(03)00064-2

[B30] Eckmann C. R. Crittenden S. L. Suh N. Kimble J. (2004). GLD-3 and control of the mitosis/meiosis decision in the germline of *Caenorhabditis elegans*. *Genetics* 168 147–160. 15454534 10.1534/genetics.104.029264 PMC1448115

[B31] Eckmann C. R. Kraemer B. Wickens M. Kimble J. (2002). GLD-3, a bicaudal-C homolog that inhibits FBF to control germline sex determination in *C. elegans*. *Dev. Cell* 3 697–710. 12431376 10.1016/s1534-5807(02)00322-2

[B32] Edwards T. A. Pyle S. E. Wharton R. P. Aggarwal A. K. (2001). Structure of Pumilio reveals similarity between RNA and peptide binding motifs. *Cell* 105 281–289. 11336677 10.1016/s0092-8674(01)00318-x

[B33] Forbes A. Lehmann R. (1998). Nanos and Pumilio have critical roles in the development and function of Drosophila germline stem cells. *Development* 125 679–690. 9435288 10.1242/dev.125.4.679

[B34] Fox P. M. Schedl T. (2015). Analysis of germline stem cell differentiation following loss of GLP-1 notch activity in *Caenorhabditis elegans*. *Genetics* 201 167–184. 10.1534/genetics.115.178061 26158953 PMC4566261

[B35] Fox P. M. Vought V. E. Hanazawa M. Lee M. H. Maine E. M. Schedl T. (2011). Cyclin E and CDK-2 regulate proliferative cell fate and cell cycle progression in the *C. elegans* germline. *Development* 138 2223–2234. 10.1242/dev.059535 21558371 PMC3091494

[B36] Friend K. Campbell Z. T. Cooke A. Kroll-Conner P. Wickens M. P. Kimble J. (2012). A conserved PUF-Ago-eEF1A complex attenuates translation elongation. *Nat. Struct. Mol. Biol.* 19 176–183. 10.1038/nsmb.2214 22231398 PMC3293257

[B37] García-Rodríguez L. J. Gay A. C. Pon L. A. (2007). Puf3p, a Pumilio family RNA binding protein, localizes to mitochondria and regulates mitochondrial biogenesis and motility in budding yeast. *J. Cell Biol.* 176 197–207. 17210948 10.1083/jcb.200606054 PMC2063939

[B38] Gerber A. P. Herschlag D. Brown P. O. (2004). Extensive association of functionally and cytotopically related mRNAs with Puf family RNA-binding proteins in yeast. *PLoS Biol.* 2:E79. 10.1371/journal.pbio.0020079 15024427 PMC368173

[B39] Gerber A. P. Luschnig S. Krasnow M. A. Brown P. O. Herschlag D. (2006). Genome-wide identification of mRNAs associated with the translational regulator PUMILIO in *Drosophila melanogaster*. *Proc. Natl. Acad. Sci. U.S.A.* 103 4487–4492. 16537387 10.1073/pnas.0509260103 PMC1400586

[B40] Glisovic T. Bachorik J. L. Yong J. Dreyfuss G. (2008). RNA-binding proteins and post-transcriptional gene regulation. *FEBS Lett.* 582 1977–1986. 10.1016/j.febslet.2008.03.004 18342629 PMC2858862

[B41] Goldstrohm A. C. Hook B. A. Seay D. J. Wickens M. (2006). PUF proteins bind Pop2p to regulate messenger RNAs. *Nat. Struct. Mol. Biol.* 13 533–539. 16715093 10.1038/nsmb1100

[B42] Hafner M. Landthaler M. Burger L. Khorshid M. Hausser J. Berninger P. (2010). Transcriptome-wide identification of RNA-binding protein and microRNA target sites by PAR-CLIP. *Cell* 141 129–141. 10.1016/j.cell.2010.03.009 20371350 PMC2861495

[B43] Hall T. M. (2016). De-coding and re-coding RNA recognition by PUF and PPR repeat proteins. *Curr. Opin. Struct. Biol.* 36 116–121. 10.1016/j.sbi.2016.01.010 26874972 PMC4757904

[B44] Hansen D. Schedl T. (2006). The regulatory network controlling the proliferation-meiotic entry decision in the *Caenorhabditis elegans* germ line. *Curr. Top. Dev. Biol.* 76 185–215. 17118267 10.1016/S0070-2153(06)76006-9

[B45] Hansen D. Wilson-Berry L. Dang T. Schedl T. (2004). Control of the proliferation versus meiotic development decision in the *C. elegans* germline through regulation of GLD-1 protein accumulation. *Development* 131 93–104. 14660440 10.1242/dev.00916

[B46] Haupt K. A. Enright A. L. Ferdous A. S. Kershner A. M. Shin H. Wickens M. (2019a). The molecular basis of LST-1 self-renewal activity and its control of stem cell pool size. *Development* 146:dev181644. 10.1242/dev.181644 31515205 PMC6826033

[B47] Haupt K. A. Law K. T. Enright A. L. Kanzler C. R. Shin H. Wickens M. (2019b). A PUF hub drives self-renewal in. *Genetics* 214 147–161. 31740451 10.1534/genetics.119.302772 PMC6944405

[B48] Henderson S. T. Gao D. Christensen S. Kimble J. (1997). Functional domains of LAG-2, a putative signaling ligand for LIN-12 and GLP-1 receptors in *Caenorhabditis elegans*. *Mol. Biol. Cell* 8 1751–1762. 9307971 10.1091/mbc.8.9.1751 PMC305734

[B49] Hirsh D. Oppenheim D. Klass M. (1976). Development of the reproductive system of *Caenorhabditis elegans*. *Dev. Biol.* 49 200–219. 943344 10.1016/0012-1606(76)90267-0

[B50] Hodgkin J. (1987). A genetic analysis of the sex-determining gene, tra-1, in the nematode *Caenorhabditis elegans*. *Genes Dev.* 1 731–745. 3428597 10.1101/gad.1.7.731

[B51] Hu S. Skelly L. E. Kaymak E. Freeberg L. Lo T. W. Kuersten S. (2019). Multi-modal regulation of *C. elegans* hermaphrodite spermatogenesis by the GLD-1-FOG-2 complex. *Dev. Biol.* 446 193–205. 10.1016/j.ydbio.2018.11.024 30599151 PMC9200065

[B52] Hubbard E. J. Korta D. Z. Dalfó D. (2013). Physiological control of germline development. *Adv. Exp. Med. Biol.* 757 101–131. 10.1007/978-1-4614-4015-4_5 22872476 PMC3760422

[B53] Hubstenberger A. Cameron C. Shtofman R. Gutman S. Evans T. C. (2012). A network of PUF proteins and Ras signaling promote mRNA repression and oogenesis in *C. elegans*. *Dev. Biol.* 366 218–231. 10.1016/j.ydbio.2012.03.019 22542599 PMC3361503

[B54] Hubstenberger A. Courel M. Bénard M. Souquere S. Ernoult-Lange M. Chouaib R. (2017). P-body purification reveals the condensation of repressed mRNA regulons. *Mol. Cell* 68 144–157.e5. 10.1016/j.molcel.2017.09.003 28965817

[B55] Hunter C. P. Kenyon C. (1996). Spatial and temporal controls target pal-1 blastomere-specification activity to a single blastomere lineage in *C. elegans* embryos. *Cell* 87 217–226. 8861906 10.1016/s0092-8674(00)81340-9

[B56] Jan E. Motzny C. K. Graves L. E. Goodwin E. B. (1999). The STAR protein, GLD-1, is a translational regulator of sexual identity in *Caenorhabditis elegans*. *EMBO J.* 18 258–269. 9878068 10.1093/emboj/18.1.258 PMC1171120

[B57] Jaramillo-Lambert A. Ellefson M. Villeneuve A. M. Engebrecht J. (2007). Differential timing of S phases, X chromosome replication, and meiotic prophase in the *C. elegans* germ line. *Dev. Biol.* 308 206–221. 17599823 10.1016/j.ydbio.2007.05.019

[B58] Jin S. W. Kimble J. Ellis R. E. (2001). Regulation of cell fate in *Caenorhabditis elegans* by a novel cytoplasmic polyadenylation element binding protein. *Dev. Biol.* 229 537–553. 11150246 10.1006/dbio.2000.9993

[B59] Kadyk L. C. Kimble J. (1998). Genetic regulation of entry into meiosis in *Caenorhabditis elegans*. *Development* 125 1803–1813. 9550713 10.1242/dev.125.10.1803

[B60] Kadyrova L. Y. Habara Y. Lee T. H. Wharton R. P. (2007). Translational control of maternal Cyclin B mRNA by Nanos in the *Drosophila* germline. *Development* 134 1519–1527. 17360772 10.1242/dev.002212

[B61] Kalchhauser I. Farley B. M. Pauli S. Ryder S. P. Ciosk R. (2011). FBF represses the Cip/Kip cell-cycle inhibitor CKI-2 to promote self-renewal of germline stem cells in *C. elegans*. *EMBO J.* 30 3823–3829. 10.1038/emboj.2011.263 21822213 PMC3173791

[B62] Kaye J. A. Rose N. C. Goldsworthy B. Goga A. L’etoile N. D. (2009). A 3′UTR pumilio-binding element directs translational activation in olfactory sensory neurons. *Neuron* 61 57–70. 10.1016/j.neuron.2008.11.012 19146813 PMC4274156

[B63] Kedde M. Van Kouwenhove M. Zwart W. Oude Vrielink J. A. Elkon R. Agami R. (2010). A Pumilio-induced RNA structure switch in p27-3′ UTR controls miR-221 and miR-222 accessibility. *Nat. Cell Biol.* 12 1014–1020. 10.1038/ncb2105 20818387

[B64] Kerins J. A. Hanazawa M. Dorsett M. Schedl T. (2010). PRP-17 and the pre-mRNA splicing pathway are preferentially required for the proliferation versus meiotic development decision and germline sex determination in *Caenorhabditis elegans*. *Dev. Dyn.* 239 1555–1572. 10.1002/dvdy.22274 20419786 PMC3097115

[B65] Kershner A. M. Kimble J. (2010). Genome-wide analysis of mRNA targets for *Caenorhabditis elegans* FBF, a conserved stem cell regulator. *Proc. Natl. Acad. Sci. U.S.A.* 107 3936–3941. 10.1073/pnas.1000495107 20142496 PMC2840422

[B66] Kershner A. M. Shin H. Hansen T. J. Kimble J. (2014). Discovery of two GLP-1/Notch target genes that account for the role of GLP-1/Notch signaling in stem cell maintenance. *Proc. Natl. Acad. Sci. U.S.A.* 111 3739–3744. 10.1073/pnas.1401861111 24567412 PMC3956202

[B67] Kimble J. Crittenden S. L. (2007). Controls of germline stem cells, entry into meiosis, and the sperm/oocyte decision in *Caenorhabditis elegans*. *Annu. Rev. Cell Dev. Biol.* 23 405–433. 17506698 10.1146/annurev.cellbio.23.090506.123326

[B68] Kimble J. Page D. C. (2007). The mysteries of sexual identity. The germ cell’s perspective. *Science* 316 400–401. 17446389 10.1126/science.1142109

[B69] Kimble J. E. White J. G. (1981). On the control of germ cell development in *Caenorhabditis elegans*. *Dev. Biol.* 81 208–219. 7202837 10.1016/0012-1606(81)90284-0

[B70] Kraemer B. Crittenden S. Gallegos M. Moulder G. Barstead R. Kimble J. (1999). NANOS-3 and FBF proteins physically interact to control the sperm-oocyte switch in *Caenorhabditis elegans*. *Curr. Biol.* 9 1009–1018. 10508609 10.1016/s0960-9822(99)80449-7

[B71] Krause M. Fire A. Harrison S. W. Priess J. Weintraub H. (1990). CeMyoD accumulation defines the body wall muscle cell fate during *C. elegans embryogenesis*. *Cell* 63 907–919. 2175254 10.1016/0092-8674(90)90494-y

[B72] Kwon S. C. Yi H. Eichelbaum K. Föhr S. Fischer B. You K. T. (2013). The RNA-binding protein repertoire of embryonic stem cells. *Nat. Struct. Mol. Biol.* 20 1122–1130. 10.1038/nsmb.2638 23912277

[B73] Lamont L. B. Crittenden S. L. Bernstein D. Wickens M. Kimble J. (2004). FBF-1 and FBF-2 regulate the size of the mitotic region in the *C. elegans germline*. *Dev. Cell* 7 697–707. 15525531 10.1016/j.devcel.2004.09.013

[B74] Lechler M. C. Crawford E. D. Groh N. Widmaier K. Jung R. Kirstein J. (2017). Reduced insulin/IGF-1 signaling restores the dynamic properties of key stress granule proteins during aging. *Cell Rep.* 18 454–467. 10.1016/j.celrep.2016.12.033 28076789 PMC5263236

[B75] Lee C. D. Tu B. P. (2015). Glucose-regulated phosphorylation of the PUF protein Puf3 regulates the translational fate of its bound mRNAs and association with RNA granules. *Cell Rep.* 11 1638–1650. 10.1016/j.celrep.2015.05.014 26051939 PMC4472502

[B76] Lee M. H. Hook B. Lamont L. B. Wickens M. Kimble J. (2006). LIP-1 phosphatase controls the extent of germline proliferation in *Caenorhabditis elegans*. *EMBO J.* 25 88–96. 16319922 10.1038/sj.emboj.7600901 PMC1351240

[B77] Lee M. H. Hook B. Pan G. Kershner A. M. Merritt C. Seydoux G. (2007a). Conserved regulation of MAP kinase expression by PUF RNA-binding proteins. *PLoS Genet.* 3:e233. 10.1371/journal.pgen.0030233 18166083 PMC2323325

[B78] Lee M. H. Ohmachi M. Arur S. Nayak S. Francis R. Church D. (2007b). Multiple functions and dynamic activation of MPK-1 extracellular signal-regulated kinase signaling in *Caenorhabditis elegans* germline development. *Genetics* 177 2039–2062. 18073423 10.1534/genetics.107.081356 PMC2219468

[B79] Lei H. Liu J. Fukushige T. Fire A. Krause M. (2009). Caudal-like PAL-1 directly activates the bodywall muscle module regulator hlh-1 in *C. elegans* to initiate the embryonic muscle gene regulatory network. *Development* 136 1241–1249. 10.1242/dev.030668 19261701 PMC2687460

[B80] Lin H. Spradling A. C. (1997). A novel group of pumilio mutations affects the asymmetric division of germline stem cells in the Drosophila ovary. *Development* 124 2463–2476. 9199372 10.1242/dev.124.12.2463

[B81] Lin K. Qiang W. Zhu M. Ding Y. Shi Q. Chen X. (2019). Mammalian Pum1 and Pum2 control body size via translational regulation of the cell cycle inhibitor Cdkn1b. *Cell Rep.* 26 2434–2450.e6. 10.1016/j.celrep.2019.01.111 30811992 PMC6444939

[B82] Liu Q. Stumpf C. Thomas C. Wickens M. Haag E. S. (2012). Context-dependent function of a conserved translational regulatory module. *Development* 139 1509–1521. 10.1242/dev.070128 22399679 PMC3308183

[B83] Lu G. Dolgner S. J. Hall T. M. (2009). Understanding and engineering RNA sequence specificity of PUF proteins. *Curr. Opin. Struct. Biol.* 19 110–115. 10.1016/j.sbi.2008.12.009 19186050 PMC2748946

[B84] Luitjens C. Gallegos M. Kraemer B. Kimble J. Wickens M. (2000). CPEB proteins control two key steps in spermatogenesis in *C. elegans*. *Genes Dev.* 14 2596–2609. 11040214 10.1101/gad.831700 PMC316992

[B85] Maheshwari R. Pushpa K. Subramaniam K. (2016). A role for post-transcriptional control of endoplasmic reticulum dynamics and function in *C. elegans* germline stem cell maintenance. *Development* 143 3097–3108. 10.1242/dev.134056 27510976 PMC6514412

[B86] Mainpal R. Priti A. Subramaniam K. (2011). PUF-8 suppresses the somatic transcription factor PAL-1 expression in *C. elegans* germline stem cells. *Dev. Biol.* 360 195–207. 10.1016/j.ydbio.2011.09.021 21968099 PMC3736097

[B87] Malik S. Jang W. Park S. Y. Kim J. Y. Kwon K. S. Kim C. (2019). The target specificity of the RNA binding protein Pumilio is determined by distinct co-factors. *Biosci. Rep.* 39:BSR20190099. 10.1042/BSR20190099 31097674 PMC6549094

[B88] Mee C. J. Pym E. C. Moffat K. G. Baines R. A. (2004). Regulation of neuronal excitability through pumilio-dependent control of a sodium channel gene. *J. Neurosci.* 24 8695–8703. 15470135 10.1523/JNEUROSCI.2282-04.2004 PMC6729971

[B89] Menichelli E. Wu J. Campbell Z. T. Wickens M. Williamson J. R. (2013). Biochemical characterization of the *Caenorhabditis elegans* FBF.CPB-1 translational regulation complex identifies conserved protein interaction hotspots. *J. Mol. Biol.* 425 725–737. 10.1016/j.jmb.2012.11.012 23159558 PMC3568192

[B90] Merritt C. Rasoloson D. Ko D. Seydoux G. (2008). 3′ UTRs are the primary regulators of gene expression in the *C. elegans* germline. *Curr. Biol.* 18 1476–1482. 10.1016/j.cub.2008.08.013 18818082 PMC2585380

[B91] Merritt C. Seydoux G. (2010). The Puf RNA-binding proteins FBF-1 and FBF-2 inhibit the expression of synaptonemal complex proteins in germline stem cells. *Development* 137 1787–1798. 10.1242/dev.050799 20431119 PMC2867315

[B92] Millonigg S. Minasaki R. Nousch M. Novak J. Eckmann C. R. (2014). GLD-4-mediated translational activation regulates the size of the proliferative germ cell pool in the adult *C. elegans* germ line. *PLoS Genet.* 10:e1004647. 10.1371/journal.pgen.1004647 25254367 PMC4177745

[B93] Morgan C. T. Lee M. H. Kimble J. (2010). Chemical reprogramming of *Caenorhabditis elegans* germ cell fate. *Nat. Chem. Biol.* 6 102–104. 10.1038/nchembio.282 20081824 PMC2808631

[B94] Morgan C. T. Noble D. Kimble J. (2013). Mitosis-meiosis and sperm-oocyte fate decisions are separable regulatory events. *Proc. Natl. Acad. Sci. U.S.A.* 110 3411–3416. 10.1073/pnas.1300928110 23401507 PMC3587202

[B95] Morris A. R. Mukherjee N. Keene J. D. (2008). Ribonomic analysis of human Pum1 reveals cis-trans conservation across species despite evolution of diverse mRNA target sets. *Mol. Cell. Biol.* 28 4093–4103. 10.1128/MCB.00155-08 18411299 PMC2423135

[B96] Morrison S. J. Kimble J. (2006). Asymmetric and symmetric stem-cell divisions in development and cancer. *Nature* 441 1068–1074. 16810241 10.1038/nature04956

[B97] Nadarajan S. Govindan J. A. Mcgovern M. Hubbard E. J. Greenstein D. (2009). MSP and GLP-1/Notch signaling coordinately regulate actomyosin-dependent cytoplasmic streaming and oocyte growth in *C. elegans*. *Development* 136 2223–2234. 10.1242/dev.034603 19502484 PMC2729341

[B98] Naudin C. Hattabi A. Michelet F. Miri-Nezhad A. Benyoucef A. Pflumio F. (2017). PUMILIO/FOXP1 signaling drives expansion of hematopoietic stem/progenitor and leukemia cells. *Blood* 129 2493–2506. 10.1182/blood-2016-10-747436 28232582 PMC5429137

[B99] Noble S. L. Allen B. L. Goh L. K. Nordick K. Evans T. C. (2008). Maternal mRNAs are regulated by diverse P body-related mRNP granules during early *Caenorhabditis elegans* development. *J. Cell Biol.* 182 559–572. 10.1083/jcb.200802128 18695046 PMC2500140

[B100] Novak P. Wang X. Ellenbecker M. Feilzer S. Voronina E. (2015). Splicing machinery facilitates post-transcriptional regulation by FBFs and other RNA-binding proteins in *Caenorhabditis elegans* germline. *G3* 5 2051–2059. 10.1534/g3.115.019315 26268245 PMC4592988

[B101] Okano H. Kawahara H. Toriya M. Nakao K. Shibata S. Imai T. (2005). Function of RNA-binding protein Musashi-1 in stem cells. *Exp. Cell Res.* 306 349–356. 15925591 10.1016/j.yexcr.2005.02.021

[B102] Olivas W. Parker R. (2000). The Puf3 protein is a transcript-specific regulator of mRNA degradation in yeast. *EMBO J.* 19 6602–6611. 11101532 10.1093/emboj/19.23.6602 PMC305854

[B103] Opperman L. Hook B. Defino M. Bernstein D. S. Wickens M. (2005). A single spacer nucleotide determines the specificities of two mRNA regulatory proteins. *Nat. Struct. Mol. Biol.* 12 945–951. 16244662 10.1038/nsmb1010

[B104] Parisi M. Lin H. (1999). The Drosophila *pumilio* gene encodes two functional protein isoforms that play multiple roles in germline development, gonadogenesis, oogenesis and embryogenesis. *Genetics* 153 235–250. 10471709 10.1093/genetics/153.1.235 PMC1460748

[B105] Pazdernik N. Schedl T. (2013). Introduction to germ cell development in *Caenorhabditis elegans*. *Adv. Exp. Med. Biol.* 757 1–16. 10.1007/978-1-4614-4015-4_1 22872472 PMC3781019

[B106] Pepper A. S. Lo T. W. Killian D. J. Hall D. H. Hubbard E. J. (2003). The establishment of *Caenorhabditis elegans* germline pattern is controlled by overlapping proximal and distal somatic gonad signals. *Dev. Biol.* 259 336–350. 12871705 10.1016/s0012-1606(03)00203-3

[B107] Porter D. F. Prasad A. Carrick B. H. Kroll-Connor P. Wickens M. Kimble J. (2019). Toward identifying subnetworks from FBF binding landscapes in. *G3* 9 153–165. 10.1534/g3.118.200300 30459181 PMC6325917

[B108] Prasad A. Porter D. F. Kroll-Conner P. L. Mohanty I. Ryan A. R. Crittenden S. L. (2016). The PUF binding landscape in metazoan germ cells. *RNA* 22 1026–1043. 10.1261/rna.055871.116 27165521 PMC4911911

[B109] Pushpa K. Kumar G. A. Subramaniam K. (2013). PUF-8 and TCER-1 are essential for normal levels of multiple mRNAs in the *C. elegans* germline. *Development* 140 1312–1320. 10.1242/dev.087833 23444359 PMC3585663

[B110] Qiu C. Bhat V. D. Rajeev S. Zhang C. Lasley A. E. Wine R. N. (2019). A crystal structure of a collaborative RNA regulatory complex reveals mechanisms to refine target specificity. *eLife* 8:e48968. 10.7554/eLife.48968 31397673 PMC6697444

[B111] Quenault T. Lithgow T. Traven A. (2011). PUF proteins: repression, activation and mRNA localization. *Trends Cell Biol.* 21 104–112. 10.1016/j.tcb.2010.09.013 21115348

[B112] Racher H. Hansen D. (2012). PUF-8, a Pumilio homolog, inhibits the proliferative fate in the *Caenorhabditis elegans* germline. *G3* 2 1197–1205. 10.1534/g3.112.003350 23050230 PMC3464112

[B113] Ratti A. Fallini C. Cova L. Fantozzi R. Calzarossa C. Zennaro E. (2006). A role for the ELAV RNA-binding proteins in neural stem cells: stabilization of Msi1 mRNA. *J. Cell Sci.* 119 1442–1452. 16554442 10.1242/jcs.02852

[B114] Rezza A. Skah S. Roche C. Nadjar J. Samarut J. Plateroti M. (2010). The overexpression of the putative gut stem cell marker Musashi-1 induces tumorigenesis through Wnt and Notch activation. *J. Cell Sci.* 123 3256–3265. 10.1242/jcs.065284 20826465

[B115] Salvetti A. Rossi L. Lena A. Batistoni R. Deri P. Rainaldi G. (2005). *DjPum*, a homologue of *Drosophila Pumilio*, is essential to planarian stem cell maintenance. *Development* 132 1863–1874. 15772127 10.1242/dev.01785

[B116] Shigunov P. Sotelo-Silveira J. Kuligovski C. De Aguiar A. M. Rebelatto C. K. Moutinho J. A. (2012). PUMILIO-2 is involved in the positive regulation of cellular proliferation in human adipose-derived stem cells. *Stem Cells Dev.* 21 217–227. 10.1089/scd.2011.0143 21649561 PMC3258435

[B117] Shin H. Haupt K. A. Kershner A. M. Kroll-Conner P. Wickens M. Kimble J. (2017). SYGL-1 and LST-1 link niche signaling to PUF RNA repression for stem cell maintenance in *Caenorhabditis elegans*. *PLoS Genet.* 13:e1007121. 10.1371/journal.pgen.1007121 29232700 PMC5741267

[B118] Sonoda J. Wharton R. P. (1999). Recruitment of Nanos to *hunchback* mRNA by Pumilio. *Genes Dev.* 13 2704–2712. 10541556 10.1101/gad.13.20.2704 PMC317116

[B119] Sonoda J. Wharton R. P. (2001). *Drosophila* brain tumor is a translational repressor. *Genes Dev.* 15 762–773. 11274060 10.1101/gad.870801 PMC312658

[B120] Sorokin E. P. Gasch A. P. Kimble J. (2014). Competence for chemical reprogramming of sexual fate correlates with an intersexual molecular signature in *Caenorhabditis elegans*. *Genetics* 198 561–575. 10.1534/genetics.114.169409 25146970 PMC4196613

[B121] Strome S. Updike D. (2015). Specifying and protecting germ cell fate. *Nat. Rev. Mol. Cell Biol.* 16 406–416. 10.1038/nrm4009 26122616 PMC4698964

[B122] Strome S. Wood W. B. (1982). Immunofluorescence visualization of germ-line-specific cytoplasmic granules in embryos, larvae, and adults of *Caenorhabditis elegans*. *Proc. Natl. Acad. Sci. U.S.A.* 79 1558–1562. 7041123 10.1073/pnas.79.5.1558 PMC346014

[B123] Stumpf C. R. Kimble J. Wickens M. (2008). A *Caenorhabditis elegans* PUF protein family with distinct RNA binding specificity. *RNA* 14 1550–1557. 10.1261/rna.1095908 18579869 PMC2491472

[B124] Suh N. Crittenden S. L. Goldstrohm A. Hook B. Thompson B. Wickens M. (2009). FBF and its dual control of gld-1 expression in the *Caenorhabditis elegans* germline. *Genetics* 181 1249–1260. 10.1534/genetics.108.099440 19221201 PMC2666496

[B125] Thompson B. E. Bernstein D. S. Bachorik J. L. Petcherski A. G. Wickens M. Kimble J. (2005). Dose-dependent control of proliferation and sperm specification by FOG-1/CPEB. *Development* 132 3471–3481. 16000383 10.1242/dev.01921 PMC1350643

[B126] Vaid S. Ariz M. Chaturbedi A. Kumar G. A. Subramaniam K. (2013). PUF-8 negatively regulates RAS/MAPK signalling to promote differentiation of *C. elegans* germ cells. *Development* 140 1645–1654. 10.1242/dev.088013 23487310 PMC3621483

[B127] Van Etten J. Schagat T. L. Hrit J. Weidmann C. A. Brumbaugh J. Coon J. J. (2012). Human Pumilio proteins recruit multiple deadenylases to efficiently repress messenger RNAs. *J. Biol. Chem.* 287 36370–36383. 10.1074/jbc.M112.373522 22955276 PMC3476303

[B128] Voronina E. Paix A. Seydoux G. (2012). The P granule component PGL-1 promotes the localization and silencing activity of the PUF protein FBF-2 in germline stem cells. *Development* 139 3732–3740. 22991439 10.1242/dev.083980 PMC3445306

[B129] Voronina E. Seydoux G. Sassone-Corsi P. Nagamori I. (2011). RNA granules in germ cells. *Cold Spring Harb. Perspect. Biol.* 3:a002774. 10.1101/cshperspect.a002774 21768607 PMC3225947

[B130] Wang C. Wilson-Berry L. Schedl T. Hansen D. (2012). TEG-1 CD2BP2 regulates stem cell proliferation and sex determination in the *C. elegans* germ line and physically interacts with the UAF-1 U2AF65 splicing factor. *Dev. Dyn.* 241 505–521. 10.1002/dvdy.23735 22275078 PMC3466600

[B131] Wang M. Ogé L. Perez-Garcia M. D. Hamama L. Sakr S. (2018). The PUF protein family: overview on PUF RNA targets, biological functions, and post transcriptional regulation. *Int. J. Mol. Sci.* 19:E410. 10.3390/ijms19020410 29385744 PMC5855632

[B132] Wang X. Mclachlan J. Zamore P. D. Hall T. M. (2002). Modular recognition of RNA by a human pumilio-homology domain. *Cell* 110 501–512. 12202039 10.1016/s0092-8674(02)00873-5

[B133] Wang X. Olson J. R. Rasoloson D. Ellenbecker M. Bailey J. Voronina E. (2016). Dynein light chain DLC-1 promotes localization and function of the PUF protein FBF-2 in germline progenitor cells. *Development* 143 4643–4653. 27864381 10.1242/dev.140921 PMC5201030

[B134] Wang X. Zamore P. D. Hall T. M. (2001). Crystal structure of a Pumilio homology domain. *Mol. Cell* 7 855–865. 11336708 10.1016/s1097-2765(01)00229-5

[B135] Wang Y. Opperman L. Wickens M. Hall T. M. (2009). Structural basis for specific recognition of multiple mRNA targets by a PUF regulatory protein. *Proc. Natl. Acad. Sci. U.S.A.* 106 20186–20191. 10.1073/pnas.0812076106 19901328 PMC2787170

[B136] Weidmann C. A. Goldstrohm A. C. (2012). *Drosophila* Pumilio protein contains multiple autonomous repression domains that regulate mRNAs independently of Nanos and brain tumor. *Mol. Cell. Biol.* 32 527–540. 10.1128/MCB.06052-11 22064486 PMC3255780

[B137] Weidmann C. A. Qiu C. Arvola R. M. Lou T. F. Killingsworth J. Campbell Z. T. (2016). Drosophila Nanos acts as a molecular clamp that modulates the RNA-binding and repression activities of Pumilio. *eLife* 5:e17096. 10.7554/eLife.17096 27482653 PMC4995099

[B138] Weidmann C. A. Raynard N. A. Blewett N. H. Van Etten J. Goldstrohm A. C. (2014). The RNA binding domain of Pumilio antagonizes poly-adenosine binding protein and accelerates deadenylation. *RNA* 20 1298–1319. 10.1261/rna.046029.114 24942623 PMC4105754

[B139] Wharton R. P. Sonoda J. Lee T. Patterson M. Murata Y. (1998). The Pumilio RNA-binding domain is also a translational regulator. *Mol. Cell* 1 863–872. 9660969 10.1016/s1097-2765(00)80085-4

[B140] Wickens M. Bernstein D. S. Kimble J. Parker R. (2002). A PUF family portrait: 3′UTR regulation as a way of life. *Trends Genet.* 18 150–157. 11858839 10.1016/s0168-9525(01)02616-6

[B141] Wreden C. Verrotti A. C. Schisa J. A. Lieberfarb M. E. Strickland S. (1997). Nanos and pumilio establish embryonic polarity in Drosophila by promoting posterior deadenylation of hunchback mRNA. *Development* 124 3015–3023. 9247343 10.1242/dev.124.15.3015

[B142] Wu J. Campbell Z. T. Menichelli E. Wickens M. Williamson J. R. (2013). A protein-protein interaction platform involved in recruitment of GLD-3 to the FBF-fem-3 mRNA complex. *J. Mol. Biol.* 425 738–754. 10.1016/j.jmb.2012.11.013 23159559 PMC3568228

[B143] Zamore P. D. Williamson J. R. Lehmann R. (1997). The Pumilio protein binds RNA through a conserved domain that defines a new class of RNA-binding proteins. *RNA* 3 1421–1433. 9404893 PMC1369583

[B144] Zanetti S. Puoti A. (2013). Sex determination in the *Caenorhabditis elegans* germline. *Adv. Exp. Med. Biol.* 757 41–69. 10.1007/978-1-4614-4015-4_3 22872474

[B145] Zhang B. Gallegos M. Puoti A. Durkin E. Fields S. Kimble J. (1997). A conserved RNA-binding protein that regulates sexual fates in the *C. elegans* hermaphrodite germ line. *Nature* 390 477–484. 9393998 10.1038/37297

[B146] Zhang M. Chen D. Xia J. Han W. Cui X. Neuenkirchen N. (2017). Post-transcriptional regulation of mouse neurogenesis by Pumilio proteins. *Genes Dev.* 31 1354–1369. 10.1101/gad.298752.117 28794184 PMC5580656

[B147] Zhu D. Stumpf C. R. Krahn J. M. Wickens M. Hall T. M. (2009). A 5′ cytosine binding pocket in Puf3p specifies regulation of mitochondrial mRNAs. *Proc. Natl. Acad. Sci. U.S.A.* 106 20192–20197. 10.1073/pnas.0812079106 19918084 PMC2787145

